# Mechanical Response and Parameter Sensitivity of Flexible Membrane Sealed Caverns for Compressed Air Energy Storage

**DOI:** 10.3390/ma18245657

**Published:** 2025-12-17

**Authors:** Lihua Hu, Jihan Liu, Zhixu Guo, Xin Liang, Liyuan Yu, Wei Li, Chengguo Hu, Yun Wu, Shihao Guo, Xuanyuan Liu, Qiming Zhou

**Affiliations:** 1 State Key Laboratory of Intelligent Construction and Healthy Operation & Maintenance of Deep Underground Engineering, China University of Mining and Technology, Xuzhou 221116, China; hulihuaxs@cumt.edu.cn (L.H.); ts24030048a31@cumt.edu.cn (J.L.); liwei9890816@cumt.edu.cn (W.L.); ts23030245p31ty@cumt.edu.cn (Q.Z.); 2 Yunlong Lake Laboratory of Deep Underground Science and Engineering, Xuzhou 221116, China; c-hucg@outlook.com

**Keywords:** compressed air energy storage (CAES), flexible membrane sealing material, mechanical response, parameter sensitivity

## Abstract

Implementing compressed air energy storage (CAES) in lined caverns provides a promising technical solution for large-scale energy storage, and the reasonable selection of sealing materials is essential for its success. Flexible membrane materials including sprayable polymers, rubber sheets, and airbags have recently been considered economical and practical sealing options. However, research on flexible membrane sealed CAES caverns remains limited, particularly regarding their mechanical response and parameter sensitivities. To address this gap, an elastic multilayer thick-walled cylinder model verified by physical model tests is proposed. Analytical solutions for the stress and displacement fields of the surrounding rock and concrete lining are derived, and a calculation scheme is designed to evaluate the influence and sensitivity of key parameters. Results indicate that under high internal pressure, both the lining and surrounding rock undergo radial compression without yielding, whereas the lining experiences adverse tensile stresses in the hoop direction. The maximum hoop tensile stress reached the order of 1~3 MPa under typical CAES operating pressures, and tensile-compressive stress transformation may occur in the lining under certain parameter combinations. Sensitivity analysis further shows that internal pressure, in situ stress, surrounding rock elastic modulus, and cavern radius are the dominant factors influencing the mechanical behavior of the system, while geometric and lining parameters have secondary but non-negligible effects. The findings provide theoretical support for the stability analysis and material design of flexible membrane sealed CAES caverns and offer useful guidance for determining allowable operating pressures and selecting lining configurations.

## 1. Introduction

With the global energy structure shifting toward low-carbon development, compressed air energy storage (CAES) has shown great potential as a large-scale physical energy storage technology [1,2,3,4]. In particular, the application of CAES in lined hard rock caverns (LRCs) constructed artificially or converted from abandoned underground mining spaces has attracted considerable attention. Compared with traditional CAES with salt caverns, CAES with LRCs possesses advantages such as the wide distribution of hard rock strata, high rock mass quality, and independence from specific geological formations, representing a major trend in the future development of large-scale CAES [5,6,7,8]. An LRC-based CAES system typically consists of a composite structure of surrounding rock, lining, and sealing layer. Among these, the proper selection of the sealing layer is not only essential for ensuring the airtightness of the storage cavern but also plays a critical role in determining the overall cost [9,10]. Consequently, it is imperative to develop a sealing technology characterized by cost-effective materials and the capability to guarantee the mechanical stability of the sealed CAES reservoir.

From the perspective of sealing structures and materials, the sealing layer of a CAES cavern can generally be classified into two types, i.e., flexible sealing and rigid sealing [10,11,12,13]. Under these two sealing conditions, the cavern structures differ, and consequently, their mechanical responses also vary. Motivated by this, researchers have conducted a series of related investigations. Commonly used rigid sealing materials include steel plates [4,7,8], airtight concrete [14], and fiberglass-reinforced plastic (FRP) [5]. Zhou et al. [15] idealized a steel plate sealed LRC as a three-layer thick-walled cylindrical structure. Based on a thermoelastic axisymmetric model, they established the governing equations for temperature and air pressure, and analytically studied the stress and displacement responses of the cavern subjected to coupled thermal and pressure loading. Similarly, Zhou et al. [16] employed numerical simulations to investigate the stress and displacement responses of the cavern under thermal–pressure coupling, as well as the influence of different sealing materials on cavern performance. Xiang et al. [8] analyzed the mechanical response and load-sharing mechanism of steel plate-sealed CAES caverns. Kim et al. [17] examined an LRC with steel plate sealing by coupling a thermodynamic multiphase flow model. They assessed the influence of the Excavation Damaged Zone (EDZ) on the stress, displacement, and stability. Rutqvist et al. [18] conducted elastic and coupled thermo–hydro–mechanical analyses using the TOUGH-FLAC simulator, revealing the stress evolution of the lining and demonstrating the role of an internal steel sealing layer in mitigating tensile stresses. In these above studies, hydrostatic in situ stresses were considered, and the solutions were obtained under axisymmetric plane conditions. More recently, Sun et al. [19] considered non-hydrostatic in situ stress conditions and derived analytical solutions for stress and displacement in single-, double-, and triple-layer CAES caverns under different lateral pressure coefficients and internal air pressures using complex variable theory. Although steel plate sealing exhibits advantages such as high strength/stability and excellent airtightness, and is therefore often recommended as a preferred sealing material for CAES caverns, the application of steel plates as sealing layers still faces several challenges, including high cost, welding difficulties, susceptibility to fatigue, buckling, and corrosion [10,11,12,13].

**Figure 1:**
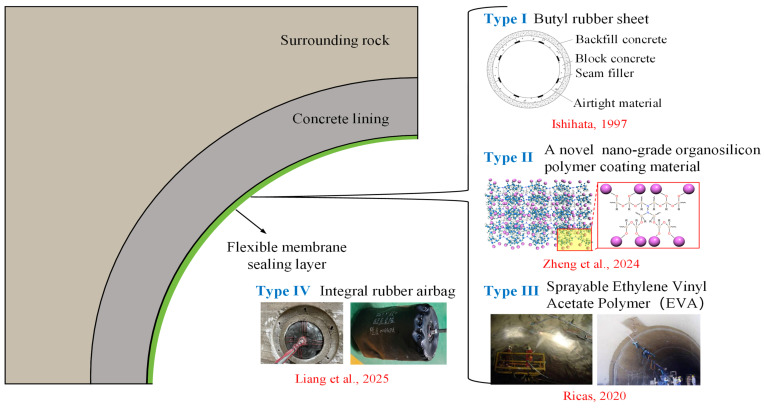
Schematic diagram of flexible membrane sealed CAES rock cavern, where four typical types of flexible membrane sealing materials are presented.

Compared with rigid sealing schemes, flexible membrane sealing technology (Figure 1) offers several advantages, including lower cost, convenient construction (e.g., by coating or spraying), and simple maintenance, rendering it a promising sealing option for CAES LRC [9,10,11,12,13]. To this end, researchers have carried out feasibility studies on using flexible membrane materials as sealing layers for CAES caverns through in situ engineering tests and laboratory-scale physical model experiments. For example, a 3 mm thick rubber sheet was adopted in the Hokkaido test cavern in Japan [13], while a 10 mm thick butyl rubber layer was applied in the Yungang mine project in China [11]. Zheng et al. [10] developed a nano-silicone polymer coating and conducted both laboratory experiments and numerical simulations, preliminarily verifying its feasibility as a sealing material for CAES caverns. More recently, Liang et al. [9], after systematically comparing the advantages and disadvantages of different sealing structures and materials, innovatively proposed the concept of using an integral rubber airbag as a sealing layer/element for CAES caverns. They carried out large-scale physical model tests and revealed the pressure distribution and deformation characteristics of the integral flexible sealing system. In addition, scholars have investigated the mechanical responses of LRCs sealed with various flexible membrane materials. Qin et al. [11,12] found that under operating pressures ranging from 4.5 to 10 MPa, the butyl rubber sealing layer was subjected to a triaxial compressive state, with a maximum von Mises stress of 1.61 MPa, which was far below its tensile strength of 8 MPa, indicating that failure would not occur. At present, CAES caverns sealed with flexible materials can be generally categorized as flexible membrane sealed caverns (Figure 1). Although many theoretical analyses, numerical simulations, laboratory-scale model tests, and in situ experiments have been conducted to study their mechanical responses, where most studies assumed that the flexible sealing layer could effectively transfer high internal pressures without adequate physical verification. In addition, the intrinsic mechanical behavior of the sealing layer itself was largely neglected. Particularly, the influence of different parameters on the mechanical response of the membrane sealing layer, particularly the stress and displacement distributions in the lining and surrounding rock structures, is still not well understood. Therefore, it is necessary to establish a rational mechanical model based on these experimental results to further investigate the stress and displacement response mechanisms of flexible membrane sealed CAES caverns.

The working performance of CAES rock caverns is influenced by multiple factors, which can be broadly categorized into three groups: geometric conditions (cavern radius and thicknesses of structural layers), loading conditions (air pressure and in situ stress), and material parameters of each structural layer (elastic modulus and Poisson’s ratio). Clarifying the influence and sensitivity of these factors on cavern responses and identifying the key parameters are fundamental for understanding the working mechanism of CAES caverns and for guiding their rational design and construction [8,20]. Xiang et al. [8] reported that the deformation modulus of the surrounding rock is the most significant factor affecting the mechanical response of the cavern, followed by cavern radius, maximum internal storage pressure, concrete lining thickness, and in situ stress. In contrast, the influence of concrete lining property was relatively minor, although increasing its strength might exacerbate cracking. Li et al. [7] analyzed the effects of convective heat transfer coefficient, thermal conductivity, charging and discharging duration, and leakage rate on the thermodynamic processes and stress responses, and found that these parameters significantly affect the distributions of temperature, pressure, and stress. Kim et al. [20] conducted a parametric sensitivity analysis focusing on the uplift of overlying strata. Currently, sensitivity analyses of influencing factors of CAES caverns are generally performed under a limited number of working conditions, where the impacts and sensitivities of parameters are qualitatively evaluated based on the calculated responses such as stress and displacement. However, studies on the effects and sensitivities of multiple variables varying continuously within their possible ranges remain insufficient. In particular, sensitivity analyses of the mechanical response of flexible membrane sealed CAES caverns have rarely been reported. Therefore, it is necessary to conduct systematic sensitivity analyses with all parameters varying continuously within their feasible ranges, so as to accurately identify the key influencing factors and thereby provide guidance for the structural optimization design of flexible membrane sealed CAES caverns.

In summary, flexible membrane sealed CAES caverns represent a highly promising technology. However, current understanding of their mechanical responses and parameter sensitivities remains limited. To address this gap, the present study establishes a mechanical analysis model for flexible membrane sealed CAES caverns, based on insights obtained from large-scale physical model experiments regarding both the intrinsic mechanical behavior of the flexible membrane sealing layer and the overall cavern response. Drawing on existing hard rock CAES projects, a computational scheme was designed that includes a set of reference parameter values and their possible variation ranges. Theoretical analyses were then carried out to investigate the distributions of radial stress, hoop stress, and radial displacement in different structural layers of the cavern under varying parameter conditions. Subsequently, based on sensitivity analysis theory and with a particular focus on the mechanical behavior at the lining–rock interface, the sensitivities of cavern mechanical responses to various parameters were systematically examined. The findings of this study are expected to provide theoretical support for the stability assessment and structural optimization design of flexible membrane sealed CAES caverns. This study is distinct from traditional analytical models for three-layer steel-lined lined rock caverns (LRC) and existing research on flexible sealing models. First, based on large-scale physical model tests, we validated that the flexible membrane can effectively transmit pressure to the lining, playing a non-negligible mechanical role. Building on this finding, we integrated the membrane behavior with the load-bearing mechanism of the lining and surrounding rock to propose the concept of a ‘flexible membrane sealed CAES cavern’. Furthermore, we established a two-layer thick-walled cylinder analytical model that specifically accounts for the load transfer characteristics of the membrane. Subsequently, a parameter sensitivity analysis was conducted to elucidate the key influencing factors and load transfer characteristics. Collectively, these contributions constitute the primary novelty of this paper.

## 2. Analytical Solution for the Mechanical Response of Flexible Membrane Sealed CAES Caverns

### 2.1. Assumptions

The analytical model established in this study adopts the following key assumptions:

(1) The initial in situ stress field is assumed to be in a hydrostatic state, where the horizontal and vertical principal stresses are equal in magnitude, and shear stress components are absent;
(2) The rock mass is treated as a homogeneous, isotropic, and linear elastic material that obeys Hooke’s law;
(3) The influences of the Excavation Damaged Zone (EDZ) and rock joints are ignored, and the surrounding rock is assumed to be a continuous medium.

The selection of these assumptions is justified by the fact that, as the initial stage of this research, simplified assumptions facilitate the effective isolation of complex interfering factors. This approach prioritizes the verification of the fundamental mechanical mechanisms governing the lining–rock interaction and simplifies the mathematical derivation to focus on core response patterns. Additionally, it aligns with the preliminary analysis requirements for the target scenarios and provides a benchmark framework for the future construction of more complex models.

However, the limitations of this model must be acknowledged. First, it is primarily applicable to scenarios characterized by an approximately hydrostatic initial stress field, high rock integrity, and no significant anisotropy. Second, it does not account for the influence of stress anisotropy on the location and magnitude of stress concentrations in the surrounding rock. Finally, by neglecting the stiffness degradation caused by the EDZ and the disruption of rock continuity by joints, the model may underestimate the magnitude of displacements and stress concentrations in actual engineering projects.

### 2.2. Evidence from Large-Scale Physical Model Tests

**Figure 2:**
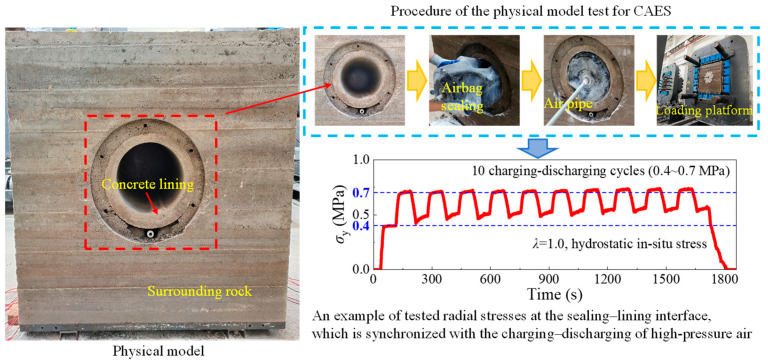
Evidence from large-scale physical model tests that the flexible membrane material is capable of sealing and transmitting high internal air pressure. The model size is 1200 × 1200 × 600 mm, with a tunnel diameter of 400 mm, lining thickness of 50 mm, and membrane thickness of 2 mm. The characteristic radii are *r*_0_ = 0.198 m, *r*_1_ = 0.20 m, *r*_2_ = 0.25 m, and *r*_3_ = 0.6 m. Material parameters: soft rock analogue (*E* = 10.7 GPa, *ν* = 0.27), concrete lining (*E* = 3.3 GPa, *ν* = 0.20), and rubber membrane (*E* = 15 MPa, *ν* = 0.47). Reinforcement was simulated using 3 mm iron mesh.

In order to verify the feasibility of flexible membrane as a sealing layer and study the mechanical response CAES caverns, the authors [9] carried out large-scale physical model tests of CAES (Figure 2). In the physical model tests, a specially manufactured 2 mm thick polyester fiber reinforced rubber airbag is used as the air storage unit, and a series of membrane pressure sensors are pasted on the outer wall (contact with the lining) of the rubber airbag. Figure 2 shows the radial stress changes at each measuring point detected by the membrane pressure sensor during cyclic charging and deflation of the high-pressure air. It can be seen from Figure 2 that the radial stress transmitted to the lining through the rubber airbag changes synchronously with the air pressure inside the chamber. Therefore, it is shown that the flexible membrane sealing layer can effectively transmit internal high air pressure. The above physical model test results can provide experimental evidence for establishing the mechanical model of flexible membrane sealed CAES caverns.

### 2.3. Mechanical Model for Flexible Membrane Sealed CAES Caverns

**Figure 3:**
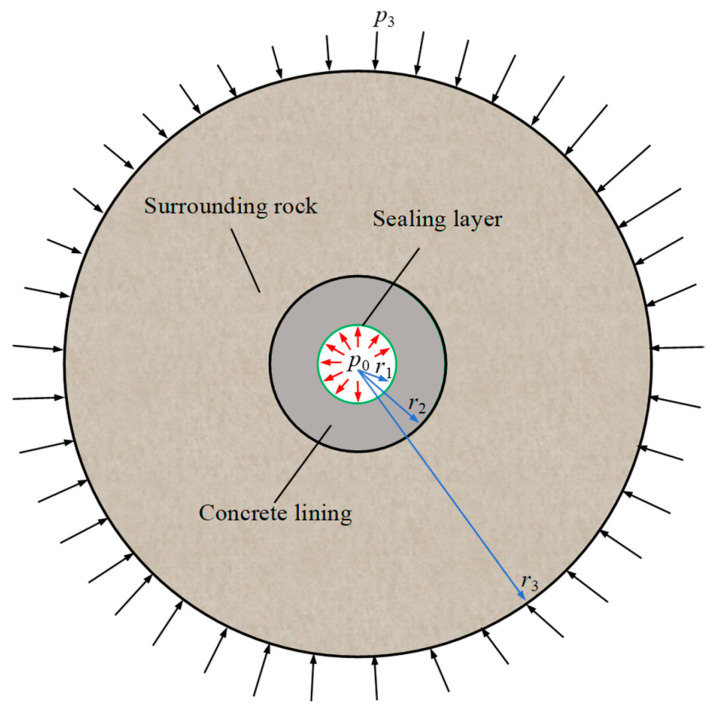
Mechanical model for a flexible membrane sealed CAES cavern, where *r*_1_ is the radius of the cavern (also the inner radius of the lining), *r*_2_ is the outer radius of the lining, *r*_3_ is the truncation boundary of the surrounding rock (*r*_3_ ≫ *r*_1_), *p*_0_ is the internal air pressure, and *p*_3_ is the in situ stress.

According to the physical model test results (Figure 2), a mechanical analysis model of the flexible membrane sealed CAES caverns was established (Figure 3). In order to reduce the cost, CAES caverns are generally built in shallow buried hard rock strata [5,6,8]. Compared with the internal high air pressure, the in situ stress is usually low, thus it is reasonable to simplify the in situ stress as uniform hydrostatic stress. Therefore, the mechanical model shown in Figure 3 is an axisymmetric plane strain problem. Furthermore, because the flexible membrane sealing layer can effectively transmit high internal pressure, and its thickness and stiffness are very low compared with other structural layers (i.e., lining and surrounding rock), the flexible membrane sealing layer only plays a sealing role, and it can be ignored in analyzing the mechanical response. Then, the structure of a flexible membrane sealed CAES cavern can be further simplified into the two-layer structure of lining–surrounding rock. Compared with the three-layer model of steel plate sealed CAES caverns [8,9,19], the two-layer structure model will greatly simplify the mechanical analysis.

According to the mechanical model shown in Figure 3, based on the theory of two-layer thick-walled cylinder, the analytical solution of the elastic mechanical response of the flexible membrane sealed CAES caverns can be obtained. It is worth noting that for the geometric dimensions of each structural layer, we ignored the thickness of the flexible film sealing layer. Assuming that the subscripts of 1, 2, and 3 represent the flexible membrane sealing layer, lining and surrounding rock, respectively, the inner and outer radii of the sealing layer are *r*_1_ (i.e., the radius of the cavern), the inner and outer radii of the lining are *r*_1_ and *r*_2_, and the inner and outer radii of the surrounding rock are *r*_2_ and *r*_3_.

### 2.4. Control Equations for Each Layer

(1) The flexible membrane sealing layer

Under operating conditions such as inflation and deflation, the flexible membrane sealing layer will show different mechanical behaviors according to the sealing material and its installation form. As shown in Figure 1, the Type I~III flexible membrane sealing layer is in close contact with the lining. Since the radial stress between the structural layers is distributed according to their relative stiffness [8,9,19], under the action of internal high air pressure, the Type I~III sealing layer itself hardly shares the radial stress, but transmits the pressure to the lining and surrounding rock. Under an almost negligible radial stress, the Type I~III flexible membrane sealing layer only undergoes minor elastic deformation. Therefore, for CAES caverns sealed by Type I~III flexible membrane, the sealing layer can be ignored in the analysis of its mechanical response, which is also the basic assumption of many previous studies [9,11,12].

However, the situation is slightly different for the Type IV flexible membrane seal layer. It is generally difficult to manufacture an airbag that exactly matches the inner diameter of the lining [9]; the outer size of the airbag is slightly smaller than the inner diameter of the lining. Therefore, for the CAES cavern sealed by Type IV flexible membrane, the sealing layer and the lining are not in close contact at first, but with a small interval Δ. In such a scenario, the Type IV flexible membrane sealing layer will undergo two different mechanical processes during charging of high-pressure air. First, when charging begins, the airbag will expand freely. In this free expansion process, the airbag is a thin-walled cylinder bearing uniform internal pressure. Due to its small thickness, the radial and hoop stresses can be considered to be approximately uniformly distributed along the thickness of the airbag, with the following stress conditions:

(1) $$\left.\begin{array}{l} \sigma_{r1}=-p_{0}\\ \sigma_{\theta 1}=\mathrm{const}=\frac{p_{0}r_{1}}{t}\\ \tau_{r\theta 1}=0 \end{array}\right\}$$

where *p*_0_ is the air pressure, *t* is the thickness of the sealing layer, *r*_1_ is the radius of the cavern, and *σ_r_*_1_, *σ_θ_*_1_ and *τ_rθ_*_1_ are the radial, hoop and shear stresses of the sealing layer, respectively. From Equation (1), it can be seen that *σ_r_*_1_/*σ_θ_*_1_ = *p*_0_·(*t*/*r*_1_), since *t*/*r*_1_ is a very small value, the radial stress can be negligible. When the displacement caused by the free expansion exceeds the interval Δ, the sealing layer will in close contact with the lining, and the subsequent mechanical behavior will be the same as that of the Type I~III flexible membrane sealing layers.

The radial displacement of the airbag during the free expansion process can be calculated according to the elastic solution of the axisymmetric plane problem:

(2) $$\left.\begin{array}{cc} \varepsilon_{\theta 1}=\frac{u_{r1}}{r}+\frac{1}{r}\frac{\partial u_{\theta 1}}{\partial \theta } & \mathrm{Geometric}\;\mathrm{equation}\\ \varepsilon_{\theta 1}=\frac{1-{\nu_{1}}^{2}}{E_{1}}\left(\sigma_{\theta 1}-\frac{\nu_{1}}{1-\nu_{1}}\sigma_{r1}\right) & \mathrm{Physical}\;\mathrm{equation}\\ u_{\theta 1}=0 & \mathrm{Axial}\;\mathrm{symmetry} \end{array}\right\}$$

where *E*_1_ and *ν*_1_ are the elastic modulus and Poisson’s ratio of the sealing layer, respectively. Thus, the maximum radial displacement of the sealing layer can be obtained from Equations (1) and (2):

(3) $${\left(u_{r1}\right)}_{\max}=u_{r1|r=r_{1}}=\frac{1-{\nu_{1}}^{2}}{E_{1}}\left(\frac{r_{1}}{t}+\frac{\nu_{1}}{1-\nu_{1}}\right)p_{0}r_{1}$$

When the following relation is satisfied:

(4) $${\left(u_{r1}\right)}_{\max}=\Delta$$

The sealing layer will complete the free expansion process. Generally speaking, the interval between the lining and the airbag sealing layer is on the scale of millimeter or centimeters, while the allowable radial displacement of the airbag under the maximum air pressure is much greater than the above interval. For example, in the physical model tests of Liang et al. [9], (*u_r_*_1_)_max_ can reach 8.1 cm. The above analysis theoretically confirms the technical feasibility of using an integral rubber airbag as a sealing layer.

Further analysis of the mechanical behavior of the flexible membrane sealing layer after radial stress transfer will reveal an interesting phenomenon. Since the inner and outer radial stresses of the flexible membrane sealing layer are equal to the air pressure, the flexible membrane sealing layer can be regarded as a thin-walled cylinder bearing the same uniform internal and external pressure, and its stress condition is:

(5) $$\left.\begin{array}{l} \sigma_{r1}=-p_{0}\\ \sigma_{\theta 1}=\frac{\left(p_{1}-p_{2}\right)r_{0}}{t}-p_{2}\\ \;\;=\frac{\left(p_{0}-p_{0}\right)r_{0}}{t}-p_{0}\\ \;\;=-p_{0}\\ \tau_{r\theta 1}=0 \end{array}\right\}$$

where *p*_1_ and *p*_2_ are the radial compressive stresses inside and outside the flexible membrane sealing layer. It can be seen from Equation (5) that when the flexible membrane sealing layer transmits air pressure, it is in a hydrostatic stress state. Qin et al. [11,12] also drew the same conclusion in the numerical study of the stress characteristics of a rubber sealing layer, and explained the reason as the Poisson’s ratio of rubber being close to the theoretical Poisson’s ratio of incompressible materials (0.5), so that the air pressure is equivalent to acting on a constrained water body, forming a three-dimensional compressive stress. Therefore, the presented theoretical analysis further reveals the force transmission mechanism and mechanical behavior of flexible membrane sealing layer.

(2) The lining and surrounding rock

After understanding the working mechanism of the flexible membrane sealing layer, the lining–surrounding rock can be regarded as a double-layer thick-walled cylindrical structure bearing the internal pressure *p*_0_ and the external in situ stress *p*_3_ (Figure 1). According to the elastic mechanics solution of the axisymmetric plane strain problem [8,9], it is easy to know that the lining and surrounding rock have the same forms of stress and displacement:

(6) $$\left.\begin{array}{l} \sigma_{ri}=\frac{A_{i}}{r^{2}}+2C_{i}\\ \sigma_{\theta i}=-\frac{A_{i}}{r^{2}}+2C_{i}\\ \mu_{ri}=\frac{1+\nu_{i}}{E_{i}}[-\frac{A_{i}}{r}+2\left(1-2\nu_{i}\right)C_{i}r] \end{array}\right\}\;(r_{i-1}\leq r\leq r_{i})$$

where *i* = 2 and 3 represent the concrete lining and surrounding rock, respectively, *r* represents the distance from the center of the cavern, *r_i_*_−1_ and *r_i_* are the inner and outer radius of the *i*-th layer, and *r*_3_ represents the truncation boundary of the surrounding rock that is far enough away from the center of the cavern. According to the previous research results [8,9], *r*_3_ = 10 *r*_1_ is desirable. *A_i_* and *C_i_* are four undetermined coefficients.

### 2.5. Analytical Solutions for the Control Equations

It can be seen from Equation (6) that in order to obtain the solution of stress and displacement field of flexible membrane sealed CAES caverns, the four undetermined coefficients need to be solved first. The internal and external load conditions, and the contact conditions between the lining and surrounding rock can be expressed as:

(7) $$\left.\begin{array}{l} {\left(\sigma_{r2}\right)}_{r=r_{1}}=-p_{0}\\ {\left(\sigma_{r3}\right)}_{r=r_{3}}=-p_{3}\\ {\left(\sigma_{r2}\right)}_{r=r_{2}}={\left(\sigma_{r3}\right)}_{r=r_{2}}\\ {\left(\mu_{r2}\right)}_{r=r_{2}}={\left(\mu_{r3}\right)}_{r=r_{2}} \end{array}\right\}$$

Then, the four governing equations related to the undetermined coefficients *A_i_* and *C_i_* can be obtained by combining Equations (6) and (7):

(8) $$\left.\begin{array}{l} \frac{A_{2}}{r_{1}^{2}}+2C_{2}=-p_{0}\\ \frac{A_{3}}{r_{3}^{2}}+2C_{3}=-p_{3}\\ \frac{A_{2}}{r_{2}^{2}}+2C_{2}=\frac{A_{3}}{r_{2}^{2}}+2C_{3}\\ \frac{1+\upsilon_{2}}{E_{2}}\left[-\frac{A_{2}}{r_{2}}+2\left(1-2\upsilon_{2}\right)C_{2}r_{2}\right]=\frac{1+\upsilon_{3}}{E_{3}}\left[-\frac{A_{3}}{r_{2}}+2\left(1-2\upsilon_{3}\right)C_{3}r_{2}\right] \end{array}\right\}$$

Let ***c*** = [*A*_2_, *C*_2_, *A*_3_, *C*_3_]^T^ denote the undetermined coefficient column vector and ***y*** = [−*p*_0_, *−p*_3_, 0, 0]^T^ denote the load column vector, then it can be obtained from Equation (8):

(9) $$\begin{array}{l} Bc=y\\ c=B^{-1}y \end{array}$$

where **B** is the relationship matrix determined by the geometry and material properties of each structural layer:

(10) $$B=\left(\begin{array}{cccc} \frac{1}{r_{1}^{2}} & 2 & 0 & 0\\ 0 & 0 & \frac{1}{r_{3}^{2}} & 2\\ \frac{1}{r_{2}^{2}} & 2 & -\frac{1}{r_{2}^{2}} & -2\\ -\frac{1+\nu_{2}}{E_{2}r_{2}} & 2\frac{\left(1+\nu_{2}\right)\left(1-2\nu_{2}\right)}{E_{2}}r_{2} & \frac{1+\nu_{3}}{E_{3}r_{2}} & -2\frac{\left(1+\nu_{3}\right)\left(1-2\nu_{3}\right)}{E_{2}}r_{2} \end{array}\right)$$

The four undetermined coefficients *A*_2_, *C*_2_, *A*_3_, and *C*_3_ can be obtained by solving the simultaneous equations expressed in Equation (9), and then the stress and displacement expressions of the concrete lining and surrounding rock can be obtained by substituting these coefficients into Equation (6). It should be noted that Equation (9) is a system of linear equations, which contains nine parameters related to the geometry, loading conditions and material properties of a CAES cavern, and its analytical expression is complicated. In this study, the symbolic equations are solved by coding with MATLAB (2024b).

## 3. Mechanical Response of CAES Caverns Under Various Influencing Factors

### 3.1. Calculation Scheme

Considering the geometric dimensions, loading conditions and material characteristics of the composite structure of a CAES cavern, and referring to the experience of projects under construction, a reference parameter combination was designed, and the possible change range of each parameter was considered to design a theoretical calculation scheme (Table 1). In Table 1, the geometric dimensions include the cavern radius and the lining thickness. The loading conditions include air pressure and initial in situ stress. The material properties include the elastic parameters (elastic modulus and Poisson’s ratio) of lining concrete and surrounding rock, where the material parameters of concrete are related to its grade (varying between C25 and C60). The change of Poisson’s ratio of surrounding rock is small, and previous studies show that it has limited influence on the mechanical response of CAES caverns, thus it is considered as a constant approximately.

### 3.2. Radial Stresses of Each Layer Under the Reference Parameters

As mentioned in Section 3.1, the reference parameter in Table 1 is a reasonable parameter design of CAES caverns. As a case, the radial stresses of lining and surrounding rock under the reference parameters are given here. Among them, the radial stress of concrete lining is:

(11) $$\sigma_{r2}=-\frac{4.2913\times 10^{8}}{r^{2}}-2.3711\times 10^{6}(\mathrm{Pa})$$

The radial stress of surrounding rock is:

(12) $$\sigma_{r3}=-\frac{3.9335\times 10^{8}}{r^{2}}-2.9301\times 10^{6}(\mathrm{Pa})$$

**Figure 4:**
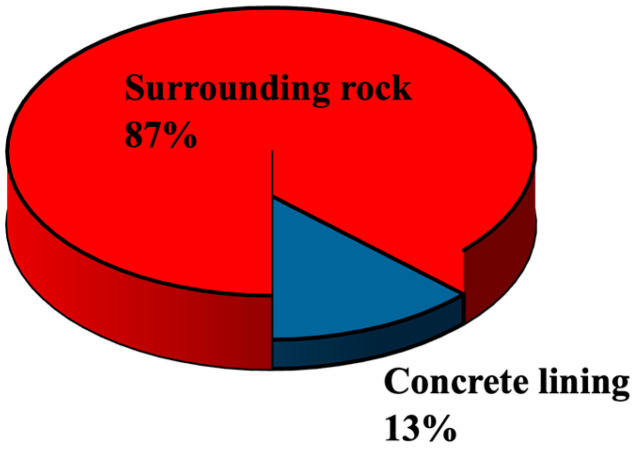
Load-sharing characteristics of a flexible membrane sealed CAES cavern under the reference parameter condition.

In addition, after obtaining the radial stress, according to the definition of Xiang et al. [8], the load sharing ratio of each structural layer can be calculated:

(13) $$\beta_{i}=\frac{\Delta \sigma_{i}}{\Delta p}\times 100\%=\left|\frac{\sigma_{i-1}-\sigma_{i}}{p_{0}-p_{3}}\right|\times 100\%$$

where *β_i_* is the load sharing ratio of each structure, Δ*σ_i_* is the radial stress difference of each structural layer, and Δ*p* is the difference between the internal pressure *P*_0_ and the in situ stress *P*_3_. The load sharing between concrete lining and surrounding rock under the reference parameter combination is shown in Figure 4. It can be seen that, consistent with previous research conclusions, the surrounding rock shares most of the load (87%) [8] and serving as a load transfer, the concrete lining shares part of the load (13%) that cannot be ignored. It can be seen that in the design concept of CAES caverns, the lining can properly share the load, which is significantly different from the traditional design concept of high-pressure underground gas storage [4,21].

### 3.3. Stress and Displacement Characteristics of Flexible Membrane Sealed CAES Caverns

Since the membrane layer only serves as a sealing function, and its mechanical behavior has been analyzed in Section 2.3, here, we focus on the distribution characteristics of radial stress, hoop stress and radial displacement of the concrete lining and surrounding rock. In order to fully understand the mechanical response of CAES caverns, we study the distribution law of stress and displacement (i.e., the functional relationship with respect to the radial coordinate *r*). Specifically, when studying the influence law of a single or multiple factors on stress and displacement, the values of other factors adopt the reference values in Table 1, so the functional relationship between stress, displacement and the studied factors can be derived according to the theoretical model:

(14) $$z=f\left(x_{i},r\right)$$

where ***z*** = [*σ_r_*, *σ_θ_*, *u_r_*] is the stress and displacement response column vector, *x_i_* is a parameter to be studied in the parameter column vector ***x*** = [*r*_1_, *t*_1_, *p*_0_, *p*_3_, *E*_2_, *E*_3_], and *r* is the distance from the center of the cavern.

#### 3.3.1. Radial Stress Distribution Characteristics

(1) Influence of geometric dimensions

**Figure 5:**
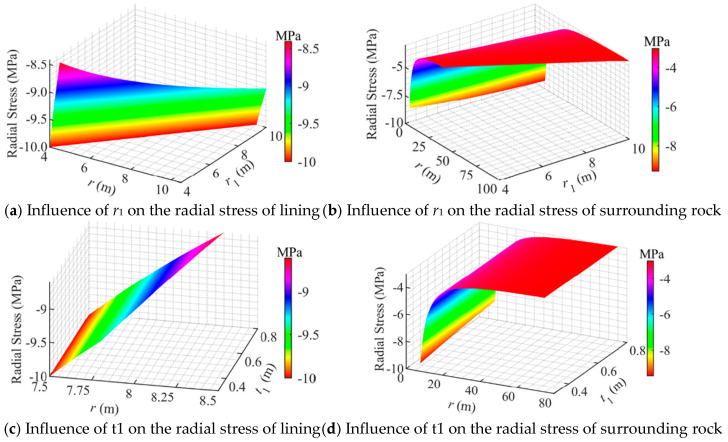
Influence of geometric dimensions on the radial stress distribution in a flexible membrane sealed CAES cavern.

Figure 5 shows the influence law of the geometric dimensions of each structural layer on the radial stresses of CAES caverns. It can be seen from Figure 5 that the radius *r*_1_ of the cravens and the thickness *t*_1_ of the lining significantly affect the radial stress of the concrete lining, while the radial stress of the surrounding rock is less affected by *t*_1_, but greatly affected by *r*_1_. In addition, the radial stress of the concrete lining layer has an approximate linear relationship with *r*_1_ and *t*_1_, while the radial stress of the surrounding rock has a nonlinear relationship with *r*_1_ and *t*_1_.

As shown in Figure 5a, the radial stress of the concrete lining decreases approximately linearly with increasing radial distance *r* when *r*_1_ varies. As *r*_1_ increases from 4 m to 10 m, the compressive radial stress at the outer boundary of the concrete lining (*r* = *r*_2_) increases from 8.4 MPa to 9.25 MPa, with an increment of approximately 9%. When *r*_1_ < 9.0 m, the radial stress at the outer boundary of the lining increases rapidly, whereas it gradually levels off as *r*_1_ > 9.0 m, indicating a stabilization trend. This demonstrates that the radial stress within the concrete lining increases with the enlargement of *r*_1_, but once *r*_1_ exceeds 9 m, the stress variation becomes negligible and the outer boundary stress stabilizes at approximately 9.2 MPa. Furthermore, the maximum radial stress in the lining remains lower than its compressive strength, implying that compressive yielding does not occur.

As shown in Figure 5b, when *r*_1_ varied, the radial stress in the surrounding rock decreased with increasing radial distance *r*, exhibiting an approximately parabolic trend and eventually declining to the in situ stress level of 3 MPa. As *r*_1_ increased from 4 m to 10 m, the compressive radial stress at the inner boundary of the surrounding rock (*r* = *r*_2_) rose from 8.3 MPa to 10 MPa, corresponding to an increase of about 17%, while the growth rate gradually decreased. This indicates that the radial stress in the surrounding rock increased with the enlargement of *r*_1_; however, when *r*_1_ exceeded 9 m, the variation tended to stabilize, and the inner boundary radial stress remained approximately constant at 9.2 MPa. Moreover, all stress values were lower than the compressive strength of the surrounding rock, implying that compressive yielding did not occur.

As shown in Figure 5c, when *t*_1_ varied, the radial stress within the concrete lining decreased approximately linearly. As *t*_1_ increased from 0.3 m to 0.8 m, the compressive radial stress at the outer boundary decreased from 9.4 MPa to 8.6 MPa, representing a reduction of about 9%, following an approximately linear trend. This indicates that increasing the lining thickness can effectively improve the radial compressive condition of the concrete lining, thereby enhancing its stability. Similarly, as shown in Figure 5d, when *t*_1_ varied, the radial stress in the surrounding rock decreased approximately parabolically and eventually approached the in situ stress. As *t*_1_ increased from 0.3 m to 0.8 m, the radial compressive stress at the inner boundary of the surrounding rock also decreased approximately linearly.

In summary, the cavern radius *r*_1_ had a noticeable effect on the radial stress distribution of the surrounding rock–lining system; however, when *r*_1_ exceeded 9 m, its influence became insignificant. Increasing the concrete lining thickness *t*_1_ can effectively reduce the radial stress within both the surrounding rock and the lining, serving as a beneficial measure for enhancing the overall stability of the CAES caverns.

(2) Influence of loading conditions

**Figure 6:**
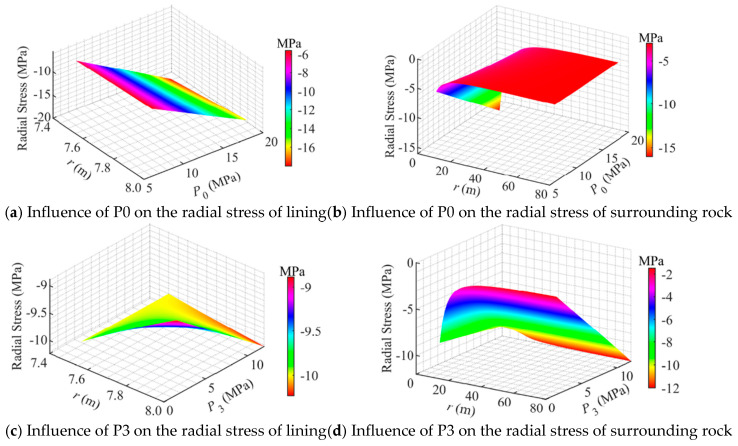
Influence of loading conditions on the radial stress distribution in a flexible membrane sealed CAES cavern.

Figure 6 illustrates the influence of loading conditions on the radial stress of CAES storage caverns. It can be observed that, within the studied ranges of air pressure *P*_0_ and in situ stress *P*_3_, the radial stress in the concrete lining is primarily governed by *P*_0_, whereas the radial stress in the surrounding rock is mainly controlled by *P*_3_.

As shown in Figure 6a, when *P*_0_ varied, the radial stress within the concrete lining decreased approximately linearly. When *P*_0_ = 6 MPa, the radial stress in the concrete lining decreased from 6 MPa at the inner boundary to 5.6 MPa at the outer boundary (*r*_2_ = 8 m), corresponding to a stress gradient of 0.8 MPa/m. When *P*_0_ = 18 MPa, the radial stress decreased from 18 MPa at the inner boundary to 16 MPa at the outer boundary, with a stress gradient of 4 MPa/m. Furthermore, as *P*_0_ increased from 6 MPa to 18 MPa, the radial stress at the outer boundary of the concrete lining exhibited an approximately linear growth trend. This indicates that *P*_0_ had a significant influence on the radial stress of the concrete lining, and increasing *P*_0_ substantially raised the stress exerted on the lining; excessive *P*_0_ may potentially induce radial compressive failure of the concrete lining.

As shown in Figure 6b, when *P*_0_ varied, the radial compressive stress in the surrounding rock exhibited an approximately parabolic decline and eventually approached the in situ stress level of 3 MPa. As *P*_0_ increased from 6 MPa to 18 MPa, the radial stress increment caused by the rise in *P*_0_ gradually diminished across the surrounding rock and approached zero near the in situ stress boundary. This indicates that the radial stress in the surrounding rock increased with increasing *P*_0_; however, compared with the concrete lining, the variation in radial stress within the surrounding rock induced by the increase in *P*_0_ was relatively less significant.

As shown in Figure 6c, when *P*_3_ < *P*_0_, the radial stress within the concrete lining decreased approximately linearly, and the stress gradient became progressively smaller. For example, when *P*_3_ = 1.5 MPa, the radial stress decreased from 10 MPa at the inner boundary to 8.9 MPa at the outer boundary; when *P*_3_ = 6 MPa, it decreased from 10 MPa to 9.5 MPa. Conversely, when the in situ stress *P*_3_ > *P*_0_, the radial stress in the concrete lining increased with increasing radial distance, also following an approximately linear trend, but with an increasing gradient. For instance, when *P*_3_ = 10.5 MPa, the radial stress rose from 10 MPa at the inner boundary to 10.04 MPa at the outer boundary, when *P*_3_ = 12 MPa, it increased from 10 MPa to 10.23 MPa. These results indicate that when the in situ stress is lower than the air pressure, an increase in *P*_3_ tends to reduce the radial stress in the concrete lining, whereas when the in situ stress exceeds the storage pressure, an increase in *P*_3_ leads to higher radial stress in the lining. Moreover, within the investigated range of *P*_3_, the radial stress in the concrete lining remains below its compressive strength, suggesting that compressive yielding does not occur.

As shown in Figure 6d, when *P*_3_ < *P*_0_ (10 MPa), the radial stress in the surrounding rock decreased with increasing radial distance, following an approximately parabolic trend, and eventually approached the in situ stress. For example, when *P*_3_ = 1.5 MPa, the radial stress decreased from 8.9 MPa at the inner boundary to 1.5 MPa at the outer boundary, when *P*_3_ = 6 MPa, it decreased from 9.5 MPa to 6 MPa. Conversely, when *P*_3_ > *P*_0_ (10 MPa), the radial stress in the surrounding rock increased with increasing radial distance, exhibiting an approximately parabolic growth and ultimately approaching the in situ stress value. For instance, when *P*_3_ = 10.5 MPa, the radial stress increased from 10.04 MPa at the inner boundary to 10.5 MPa at the outer boundary; when *P*_3_ = 12 MPa, it increased from 10.23 MPa to 12 MPa. These results indicate that when the in situ stress is lower than the air pressure, an increase in *P*_3_ significantly reduces the radial stress in the surrounding rock, whereas when the in situ stress exceeds the storage pressure, an increase in *P*_3_ markedly increases the radial stress. Therefore, under conditions where the in situ stress does not exceed the air pressure, an increase in *P*_3_ is beneficial to the stability of the storage cavern. Similarly, the radial stress in the surrounding rock remained below its compressive strength, indicating that compressive yielding did not occur.

In summary, the radial stress of the concrete lining is primarily influenced by air pressure, whereas the radial stress of the surrounding rock is governed by the combined effects of air pressure and in situ stress. This is because a load predominantly affects the physical body in its immediate vicinity [9]. This finding provides important theoretical guidance for designing the air pressure and selecting an appropriate in situ stress condition (e.g., cavern depth) for CAES caverns.

(3) Influence of material properties

**Figure 7:**
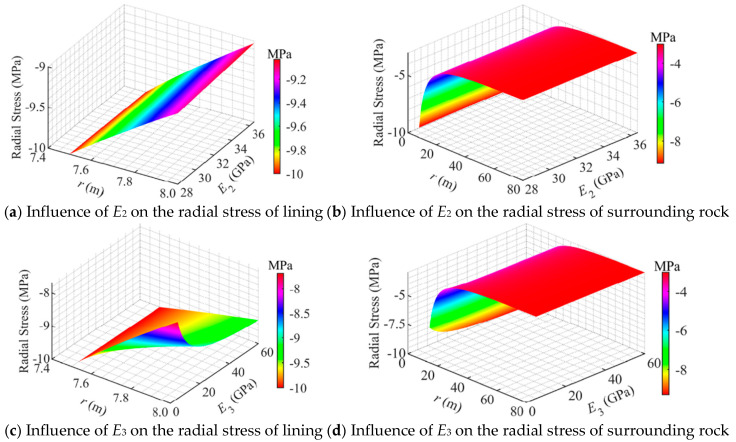
Influence of material properties on the radial stress distribution in a flexible membrane sealed CAES cavern.

Figure 7 illustrates the influence of material parameters on the radial stress. It can be observed that the elastic modulus of the surrounding rock (*E*_3_) has a significant effect on the radial stress in both the concrete lining and the surrounding rock. This finding is consistent with previous studies, which identified *E*_3_ as a key factor governing stress distribution in CAES caverns [8,9,19].

As shown in Figure 7a, when the elastic modulus of the concrete lining (*E*_2_) varied, the radial stress within the lining decreased approximately linearly with increasing radial distance. As *E*_2_ increased from 28 GPa to 36.5 GPa, the radial stress at the outer boundary of the concrete lining decreased from 9.1 MPa to 9.0 MPa, corresponding to a reduction of about 1%. This indicates that increasing *E*_2_ of the concrete lining has a very limited effect on reducing its radial stress.

As shown in Figure 7b, under different values of *E*_2_, the radial stress in the surrounding rock exhibited a consistent variation pattern, i.e., decreasing approximately parabolically from the inner boundary to the in situ stress of 3 MPa. As *E*_2_ increased from 28 GPa to 36.5 GPa, the radial stress at all positions within the surrounding rock remained nearly unchanged, indicating that the influence of *E*_2_ on the radial stress of the surrounding rock is negligible.

As shown in Figure 7c, the radial stress in the concrete lining increased significantly with the increase in *E*_3_ (5–60 GPa). The radial stress at the outer boundary of the concrete lining rose from 7.7 MPa to 9.3 MPa, corresponding to an increase of approximately 17%. This indicates that increasing *E*_3_ can effectively enhance the radial stress of the concrete lining. When *E*_3_ ranges from 5 GPa to 20 GPa, the radial stress in the concrete lining increases rapidly, when *E*_3_ is between 20 GPa and 40 GPa, the increase becomes more gradual, and when *E*_3_ exceeds 40 GPa, the radial stress tends to stabilize. These results suggest that the influence of *E*_3_ on the radial stress of the concrete lining exhibits a threshold effect.

Similar to Figure 7b,d, it shows that as *E*_3_ increases (5–60 GPa), the radial stress in the surrounding rock decreases parabolically with increasing radial distance, from the inner boundary to the in situ stress of 3 MPa. With increasing *E*_3_, the radial stress within the surrounding rock exhibited a variation trend similar to that observed in the concrete lining: when *E*_3_ ranged from 5 GPa to 20 GPa, the radial stress increased significantly, when *E*_3_ was between 20 GPa and 40 GPa, the increase became more gradual, and when *E*_3_ exceeded 40 GPa, the radial stress increment tended to stabilize. This indicates that *E*_3_ has a pronounced influence on the radial stress of the surrounding rock, and this influence also exhibits a clear threshold effect.

#### 3.3.2. Hoop Stress Distribution Characteristics

(1) Influence of geometric dimensions

**Figure 8:**
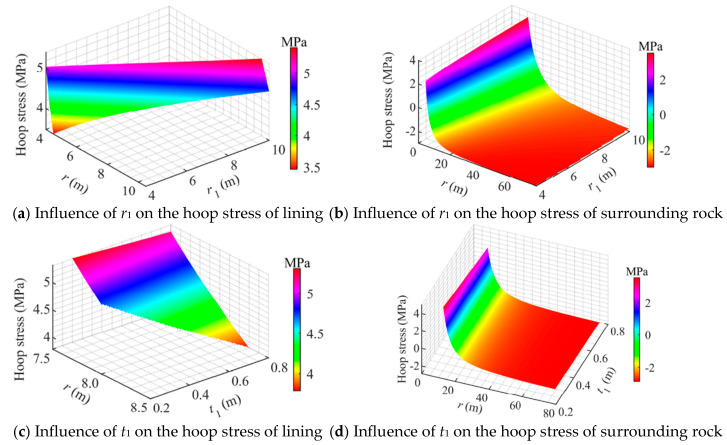
Influence of geometric dimensions on the hoop stress distribution in a flexible membrane sealed CAES cavern.

Figure 8 illustrates the influence of geometric dimensions on the hoop stresses. It can be observed that variations in the cavern radius *r*_1_ and concrete lining thickness *t*_1_ had a limited effect on the hoop stress of the concrete lining, which exhibited an approximately linear relationship with both *r*_1_ and *t*_1_. In contrast, the hoop stress in the surrounding rock showed a nonlinear relationship with *r*_1_ and *t*_1_.

As shown in Figure 8a, when *r*_1_ varied within 4.0–10.0 m, the maximum hoop tensile stress in the concrete occurred at the inner surface (*r* = *r*_1_), while the hoop tensile stress at the outer boundary of the lining (*r* = *r*_2_) rose from 3.5 MPa to 5.2 MPa. The increase was relatively rapid at smaller radii, particularly when *r*_1_ < 9.0 m, while for *r*_1_ exceeding 9.0 m, the growth of hoop stress gradually slowed and tended to stabilize. These results indicate that enlarging the cavern radius significantly increases the hoop tensile stress at the lining’s inner surface and at the lining–rock interface, thereby raising the risk of tensile cracking in the lining.

As shown in Figure 8b, the hoop stress in the surrounding rock transfers from tensile near the lining to compressive at greater radial distances, eventually approaching the in situ stress. As *r*_1_ increased from 4 m to 10 m, the hoop tensile stress at the inner boundary of the surrounding rock (*r* = *r*_2_) rose from 2.4 MPa to 3.5 MPa. However, this increase became significantly slower when *r*_1_ approached 9 m, indicating that the amplification effect of the cavern radius on the tensile stress at the rock–lining interface exhibits a diminishing trend.

As shown in Figure 8c, with increasing *t*_1_, the hoop stress within the lining showed a slight decreasing trend: the tensile stress at the outer boundary of the lining decreased marginally from 5.3 MPa to 5.1 MPa. This indicates that increasing the lining thickness can slightly mitigate the peak hoop tensile stress in the lining; however, this effect is limited, suggesting that simply increasing the lining thickness is not sufficient to effectively reduce the high tensile stress caused by high internal pressure or large cavern radius.

As shown in Figure 8d, with increasing *t*_1_, the hoop stress in the surrounding rock transfers from tensile near the lining to compressive at greater distances. Notably, the hoop tensile stress at the inner boundary of the surrounding rock (*r* = *r*_2_) decreases linearly with increasing *t*_1_. This indicates that increasing the lining thickness can partially mitigate the tensile stress in the surrounding rock and reduce the extent of the tensile stress zone, which is beneficial for enhancing the stability of the surrounding rock.

(2) Influence of loading conditions

**Figure 9:**
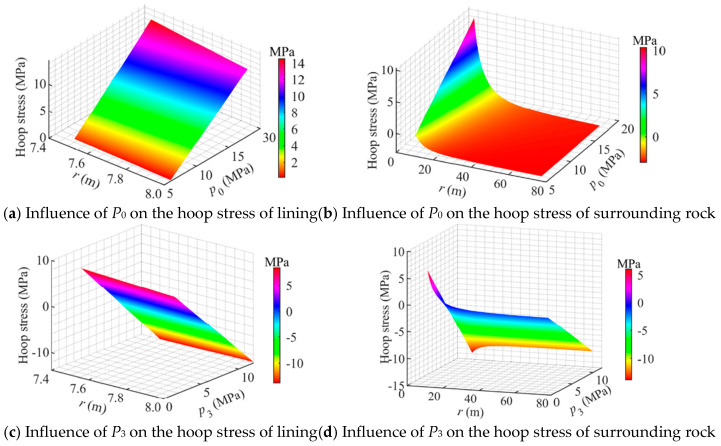
Influence of loading conditions on the hoop stress distribution in a flexible membrane sealed CAES cavern.

Figure 9 illustrates the influence of loading conditions. It can be observed that the hoop stress in the concrete lining varies linearly with both the internal pressure *P*_0_ and the in situ stress *P*_3_, whereas the hoop stress in the surrounding rock exhibits a nonlinear relationship with *P*_0_ and *P*_3_.

As shown in Figure 9a, *P*_0_ has a significant effect on the hoop stress of the concrete lining. When *P*_0_ varied from 6 MPa to 18 MPa, the hoop tensile stress at the outer boundary of the lining (*r* = *r*_2_) increased from 0.2 MPa to 12 MPa, exhibiting an approximately linear relationship. These results indicate that under high internal pressure, the hoop tensile stress in the lining can rapidly approach or even exceed the tensile strength of concrete, posing a considerable risk of cracking.

As shown in Figure 9b, as *P*_0_ increased from 6 MPa to 18 MPa, the hoop tensile stress at the inner boundary of the surrounding rock (*r* = *r*_2_) rose from 0.3 MPa to 10 MPa. Moving away from the lining, the hoop tensile stress in the surrounding rock gradually decreased and eventually approached the in situ compressive stress.

As shown in Figure 9c, as *P*_3_ increased from a lower to a higher value, the hoop stress at the outer boundary of the lining can change from tensile to compressive. Under *P*_3_ = 6 MPa, the hoop tensile stress at *r* = *r*_2_ was approximately 1.7 MPa, whereas when *P*_3_ = 12 MPa, the same location experienced a compressive stress of 13 MPa. This indicates that when the external in situ stress approaches or exceeds the internal pressure, both the sign and magnitude of the circumferential stress field undergo fundamental changes, which is beneficial for the safety of the storage cavern.

As shown in Figure 9d, as *P*_3_ increased from 1.5 MPa to 12 MPa, the hoop stress at the inner boundary of the surrounding rock (*r* = *r*_2_) transferred from 6 MPa (tensile) to 12 MPa (compressive). This indicates that increasing the in situ stress *P*_3_ can counteract the hoop tensile stress induced by internal pressure, which is beneficial for inhibiting the development of cracks in the lining.

(3) Influence of material properties

**Figure 10:**
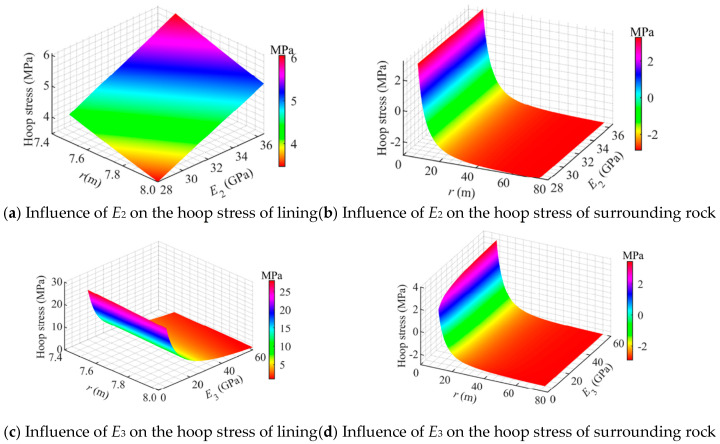
Influence of material properties on the hoop stress distribution in a flexible membrane sealed CAES cavern.

Figure 10 illustrates the influence of material parameters. It can be observed that the hoop stress in the concrete lining is sensitive to the surrounding rock elastic modulus *E*_3_, while the hoop stress in the surrounding rock is primarily governed by its own modulus *E*_3_ and increases gradually as *E*_3_ rises.

As shown in Figure 10a, when *E*_2_ increased from 28 GPa to 36.5 GPa, the hoop tensile stress at the outer boundary of the lining rose from 3.5 MPa to 5.0 MPa. Therefore, increasing the stiffness (grade) of the lining concrete slightly enhances its tensile strength, but also it amplifies the peak tensile stress, and induced tensile stress increase exceeds that of its strength, thereby elevating the risk of cracking in the lining.

As shown in Figure 10b, as *E*_2_ increased from 28 GPa to 36.5 GPa, the hoop stress at the inner boundary of the surrounding rock (*r* = *r*_2_) remained nearly unchanged, decreasing slightly from 3.2 MPa to 3.1 MPa. This indicates that increase in *E*_2_ has little effect on the hoop stress of the surrounding rock.

As shown in Figure 10c, with increasing *E*_3_ (5–60 GPa), the hoop stress in the concrete lining continuously decreases. The hoop tensile stress at the outer boundary of the lining (*r* = *r*_2_) drops from 25 MPa to 1.2 MPa. This indicates that increasing *E*_3_ can effectively reduce the hoop tensile stress in the concrete lining.

As shown in Figure 10d, with increasing *E*_3_ (5–60 GPa), the hoop tensile stress at the inner boundary of the surrounding rock (*r* = *r*_2_) rises from 1.8 MPa to 3.4 MPa.

#### 3.3.3. Radial Displacement Distribution Characteristics

(1) Influence of geometric dimensions

**Figure 11:**
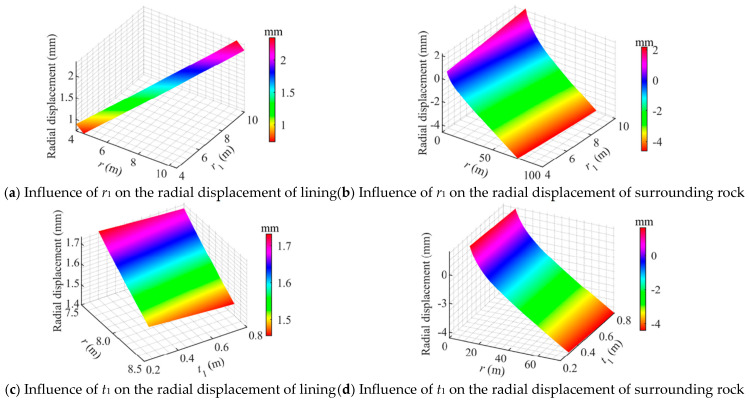
Influence of geometric dimensions on the radial displacement of a flexible membrane sealed CAES cavern.

Figure 11 illustrates the influence of cavern radius *r*_1_ and lining thickness *t*_1_ on the radial displacements. As shown, the radial displacement of the concrete lining varies linearly with both *r*_1_ and *t*_1_, whereas the radial displacement of the surrounding rock exhibits a nonlinear relationship with *r*_1_ and *t*_1_.

As shown in Figure 11a, when *r*_1_ increased from 4 m to 10 m, the radial displacement of the lining increased approximately linearly. The maximum radial displacement occurred at the inner surface (*r* = *r*_1_). The radial displacement increase rate was more pronounced when *r*_1_ < 8.5 m and gradually slowed down when *r*_1_ > 9.0 m. These results indicate that enlarging the cavern radius significantly increases the outward deformation of the lining, which can adversely affect the fit of the sealing layer and the overall stability of the lining.

As shown in Figure 11b, the radial displacement distribution of the surrounding rock exhibits a distinct directional reversal as *r*_1_ increases. Near the lining interface (*r* = *r*_2_), the surrounding rock undergoes outward displacement, while in regions farther from the lining, the displacement reverses direction (toward the cavern center). As *r*_1_ increases, the outward displacement at the inner boundary becomes more pronounced, and the displacement reversal point shifts further outward.

As shown in Figure 11c, when *t*_1_ increased from 0.3 m to 0.8 m, the radial displacement of the lining decreased. At the inner surface, the maximum displacement decreased from 2.3 mm to 1.7 mm, while the outer surface displacement decreased from 1.5 mm to 1.4 mm. This indicates that increasing the lining thickness effectively enhances its overall stiffness and significantly reduces its expansion deformation.

As shown in Figure 11d, with the increase in *t*_1_, the outward displacement at the inner boundary of the surrounding rock decreases significantly, while the far-field displacement remains almost unaffected.

(2) Influence of loading conditions

**Figure 12:**
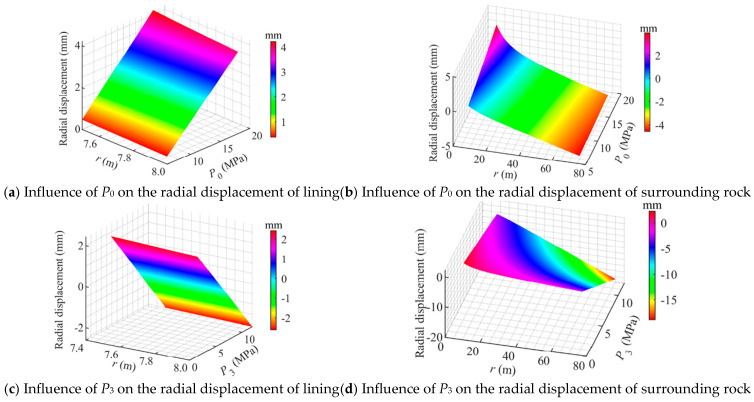
Influence of loading conditions on the radial displacement of a flexible membrane sealed CAES cavern.

The effects of the internal pressure *P*_0_ and in situ stress *P*_3_ on the radial displacement are illustrated in Figure 12. It can be observed that the radial displacement of the concrete lining varies linearly with both *P*_0_ and *P*_3_, while the radial displacement of the surrounding rock exhibits a nonlinear relationship with these parameters.

Figure 12a shows that *P*_0_ has a significant amplifying effect on the radial displacement of the lining. As *P*_0_ increased from 6 MPa to 18 MPa, the radial displacement at the inner surface of the lining increased from 0.4 mm to 4.5 mm, while that at the outer surface increased from 0.3 mm to 3.9 mm.

Figure 12b illustrates that with the increase in *P*_0_, a noticeable outward displacement occurs in the surrounding rock near the lining, where the displacement at the inner boundary rose from 0.9 mm to 5.0 mm. However, the displacement of the surrounding rock decreases rapidly with distance from the lining. This indicates that the influence of internal pressure is confined to a limited region adjacent to the lining, while the far-field displacement of the surrounding rock remains primarily governed by the in situ stress. Such deformation behavior suggests that under high-pressure CAES operating conditions, particular attention should be paid to monitoring the local deformation of the surrounding rock near the lining.

Figure 12c shows that the increase in *P*_3_ effectively suppresses the outward deformation of the lining. As *P*_3_ increased from 1.5 MPa to 12 MPa, the radial displacement at the inner surface of the lining continuously decreased, indicating that a higher confinement contributes to enhancing the overall structural stability of the CAES cavern.

Figure 12d shows that the outward displacement of the surrounding rock decreases continuously with increasing *P*_3_. The displacement at the inner boundary decreased from 0.6 mm to 0.2 mm, indicating that increasing the burial depth of the CAES cavern (and thus the in situ stress) can effectively reduce the radial deformation of the cavern.

(3) Influence of material properties

**Figure 13:**
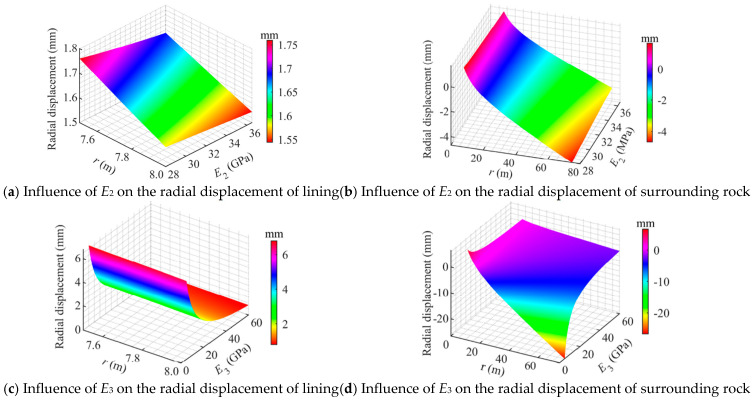
Influence of material properties on the radial displacement of a flexible membrane sealed CAES cavern.

Figure 13 illustrates the influence of the lining elastic modulus *E*_2_ and the surrounding rock elastic modulus *E*_3_ on the radial displacements. As shown in Figure 13, with the increase in *E*_2_, the deformation of the lining decreased significantly, while *E*_3_ exerts a stronger effect on the overall displacement field of the cavern. This indicates that a stiffer surrounding rock can effectively constrain the expansion deformation of the CAES cavern.

Figure 13a shows that *E*_2_ has a significant effect on radial displacement. As *E*_2_ increased from 28 GPa to 36.5 GPa, the radial displacement at the inner surface of the lining decreased from 1.75 mm to 1.69 mm, while the outer surface displacement decreased from 1.61 mm to 1.50 mm. This deformation behavior of the lining is of great importance for reducing the opening of the sealing layer.

Figure 13b shows that variations in *E*_2_ have a relatively weak effect on the displacement of the surrounding rock. The radial displacement at the inner boundary of the surrounding rock exhibits only a slight decrease.

Figure 13c indicates that *E*_3_ has a pronounced weakening effect on the radial displacement of the lining. As *E*_3_ increased from 5 GPa to 60 GPa, the radial displacement at the inner boundary of the lining decreased markedly from 6.3 mm to 1.7 mm.

Figure 13d shows that an increase in *E*_3_ leads to a significant reduction in the radial displacement on both the inner and outer sides of the surrounding rock. The displacement near the lining side (*r* = *r*_2_) decreased from 3 mm to 1 mm, while that farther from the lining side increased from −2.5 mm to 1 mm, indicating a substantial reduction in the overall deformation amplitude. These findings suggest that a stiffer surrounding rock not only effectively constrains the expansion of the lining but also mitigates its own deformation, which is highly beneficial for the stability of flexible membrane sealed CAES caverns.

## 4. Parameter Sensitivity Analysis

### 4.1. Description of the Method

As previously mentioned, a flexible membrane sealed CAES cavern involves many parameters (Table 1), and the influence of each parameter on its mechanical response varies. Therefore, in order to rationally design a CAES cavern, it is necessary to quantitatively assess the impact of each parameter, i.e., to conduct a parameter sensitivity analysis. Indeed, parameter sensitivity analysis is an important research topic for CAES caverns. In previous studies, researchers typically varied a single factor, selected several discrete parameter combinations, and calculated the resulting changes in a mechanical response indicator (such as hoop stress, load-sharing ratio, or uplift displacement) to evaluate the sensitivity of that parameter [8,20]. Although this approach is simple and intuitive, it lacks statistical rigor and may lead to the misjudgment of parameter sensitivity if the range or combinations of parameters are inadequately chosen. Therefore, it is essential to adopt a parameter sensitivity analysis method in which a large number of sample points are selected across the possible variation range of each factor, and the sensitivity is quantitatively evaluated based on the statistical results of changes in a series of indicators caused by variations in each parameter.

To perform a parameter sensitivity analysis of the mechanical response of flexible membrane sealed CAES caverns based on sensitivity analysis and statistical theory, it is first necessary to construct a dataset, which includes both input and output datasets. The input dataset represents the parameter space of the CAES cavern and consists of parameter vectors along with as many samples as possible within their respective variation ranges:

(15) $$x={\left[r_{1},t_{1},p_{0},p_{3},E_{2},E_{3}\right]}^{T}$$

$x_{j}\in x$ with its variation range listed in Table 1.

The output dataset consists of selected mechanical response indicators and their corresponding values. Since the stress and displacement in a flexible membrane sealed CAES cavern are non-uniformly distributed, some characteristic positions in the structure of a CAES cavern were selected for analysis. In this study, the lining–surrounding rock interface (*r* = *r*_2_) is selected as a characteristic contact surface (denoted as interface I). For the lining, at interface I, its stress or displacement is denoted with subscript 2, while for the surrounding rock, its stress or displacement is denoted with the subscript 3. Thus, the set of sensitivity analysis indicators can be expressed as:

(16) $$y={\left[\sigma_{I,r2},\sigma_{I,r3},\sigma_{I,\theta 2},\sigma_{I,\theta 3},u_{I,r2},u_{I,r3}\right]}^{T}$$

In the above expression, $\sigma_{I,r2}$ represents the radial stress of the lining at interface I, and the meanings of the other parameters follow similarly.

Next, the sample data in the input dataset and the corresponding data for the sensitivity analysis indicator set (i.e., the output dataset) are constructed. For any given parameter $x_{j}\in x$, let its value range be $\left[x_{j,\min},x_{j,\max}\right]$. Within this range, *n* (=1000) sample points are randomly generated using the Monte Carlo method:

(17) $$x_{j,i}=x_{j,\min}+(x_{j,\max}-x_{j,\min})\cdot \xi_{i},\;i=1,2,\ldots ,1000$$

where $\xi_{i}∼U\left(0,1\right)$ represents a uniformly distributed random number on the interval [0, 1]. Only the parameters $x_{j}$ for which sensitivity is to be analyzed are sampled according to Equation (17), while the remaining parameters are set to their reference values (Table 1) to obtain the input dataset sample points. By substituting the parameter samples generated from Equation (17) into the theoretical analysis model, the corresponding output dataset can be calculated:

(18) $$y={\left[\sigma_{I,r2},\sigma_{I,r3},\sigma_{I,\theta 2},\sigma_{I,\theta 3},u_{I,r2},u_{I,r3}\right]}^{T}=f\left(x_{j}\right)$$

where ***f*** represents the functional relationship between the stress or displacement at interface I and the corresponding parameter. Furthermore, the sensitivity of a parameter is quantitatively evaluated using the normalized standard deviation (NSD) of the output data as the indicator:

(19) $$S_{j}=\frac{{\delta (y)|}_{x_{j}}}{\left|{\mu (y)|}_{x_{j}}\right|}$$

where ${\delta (y)|}_{x_{j}}$ and ${\mu (y)|}_{x_{j}}$ are the standard deviation and mean of the output induced by the variation of parameter $x_{j}$ over its range, respectively. The indicator $S_{j}$ (referred to as the sensitivity coefficient) reflects the relative magnitude of the output variation caused by perturbations in the input parameter. Being dimensionless, it can be directly used to compare sensitivities among different physical quantities. A larger $S_{j}$ indicates a higher sensitivity of parameter $x_{j}$.

It should be noted that the parameter sensitivity coefficient $S_{j}$ in Equation (19) does not explicitly decompose the interaction effects among multiple factors. However, it can reflect the relative importance of a single parameter within its variation range. Therefore, the parameter sensitivity analysis method employed in this study can be used for the preliminary ranking and screening of the importance of various factors in a CAES cavern. For more detailed characterization of the interactions among multiple factors, higher-order variance decomposition methods, such as the Sobol sensitivity analysis, can be further applied [21,22,23,24,25,26,27,28].

To quantitatively characterize the influence of input parameters, this study employed the Normalized Standard Deviation (NSD) method. It should be noted that NSD is a screening approach based on the one-factor-at-a-time (OAT) strategy, aimed at providing a preliminary ranking of parameter importance rather than a rigorous global sensitivity analysis (GSA). Although this method does not capture parameter interactions, its ranking trends generally align with variance-based global methods (e.g., first-order Sobol indices), making it a suitable and efficient tool for preliminary sensitivity identification in engineering analysis.

### 4.2. Analysis Results

**Figure 14:**
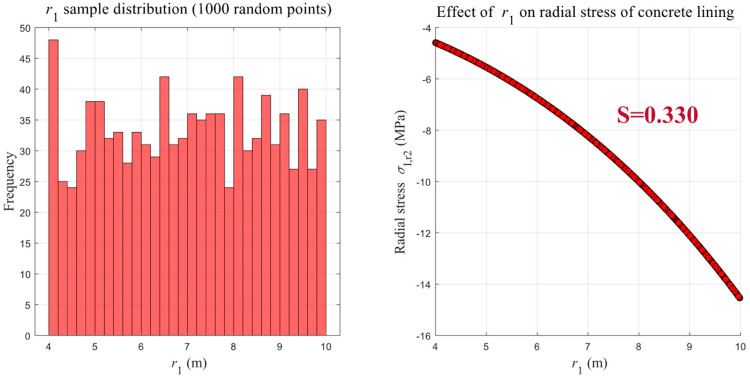
Sampling points distribution of *r*_1_ and its relationship with radial stress *σ*_I_, *_r_*_1_.

Taking the sensitivity analysis of cavern radius *r*_1_ on the lining radial stress as an example, the specific implementation process described in Section 4.1 can be illustrated intuitively. First, 1000 sample points are uniformly generated within the range of *r*_1_ (4–10 m), and their distribution is analyzed (Figure 14). Based on this, each sample is substituted into Equation (6) to calculate the corresponding radial stress $\sigma_{I,r2}$, and the relationship curve between *r*_1_ and $\sigma_{I,r2}$ is plotted (Figure 14). Subsequently, according to Equations (18) and (19), the sensitivity coefficient of *r*_1_ with respect to $\sigma_{I,r2}$ is calculated as S = 0.330.

**Figure 15:**
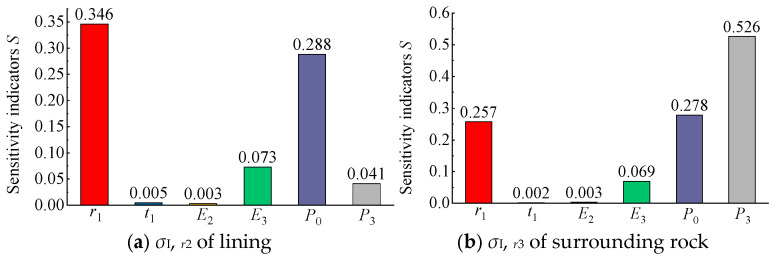
Sensitivities of different factors on the lining–surrounding rock interface radial stresses.

**Figure 16:**
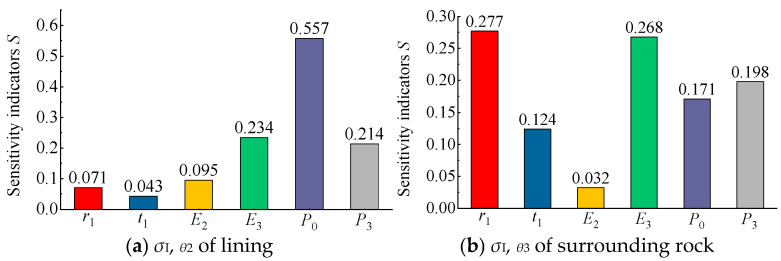
Sensitivities of different factors on the lining–surrounding rock interface hoop stresses.

**Figure 17:**
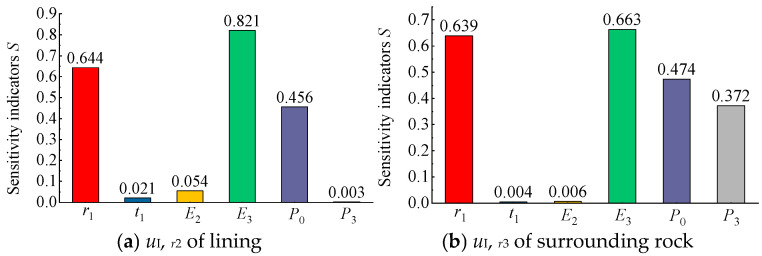
Sensitivities of different factors on the lining–surrounding rock interface radial displacements.

Based on the above method, the sensitivity coefficients of each factor for different mechanical response indicators can be calculated, allowing for a quantitative assessment of their relative sensitivities. For intuitive visualization, the calculated sensitivity coefficients of each factor with respect to the radial stress, hoop stress, and radial displacement are presented in bar charts (Figure 15, Figure 16 and Figure 17).

As shown in Figure 15a, for the lining radial stress $\sigma_{I,r2}$, the cavern radius *r*_1_ exhibited the highest sensitivity, followed closely by the air pressure *P*_0_. In contrast, the surrounding rock elastic modulus *E*_3_ and in situ stress *P*_3_ showed relatively low sensitivities, while the lining thickness *t*_1_ and elastic modulus *E*_2_ had negligible effects. Similarly, Figure 15b indicates that for the surrounding rock radial stress $\sigma_{I,r3}$, the sensitivities of the factors decreased in the order of *P*_3_, *P*_0_, *r*_1_, *E*_3_, *E*_2_, and *t*_1_. Overall, for the radial stress of the CAES cavern, the loading conditions (*P*_0_ and *P*_3_) were the dominant influencing factors, the cavern radius *r*_1_ also had a notable effect, while the lining thickness *t*_1_ and elastic modulus *E*_2_ were almost negligible.

As shown in Figure 16a, for the hoop stress of the concrete lining $\sigma_{I,\theta 2}$, the air pressure *P*_0_ exhibited the highest sensitivity, while the surrounding rock elastic modulus *E*_3_ and the in situ stress *P*_3_ also showed relatively high and comparable sensitivities. In contrast, the sensitivities of the lining elastic modulus *E*_2_, lining thickness *t*_1_, and cavern radius *r*_1_ were relatively low. As illustrated in Figure 16b, for the hoop stress of the surrounding rock $\sigma_{I,\theta 3}$, the sensitivities of the influencing factors decreased in the order of *r*_1_, *E*_3_, *P*_3_, *P*_0_, *t*_1_, and *E*_2_. Compared with the radial stress sensitivity results shown in Figure 15, the sensitivity patterns of the hoop stress for the lining and surrounding rock differed significantly. Nevertheless, the air pressure *P*_0_ and the surrounding rock elastic modulus *E*_3_ remained the most influential parameters.

As shown in Figure 17a, for the radial displacement of the concrete lining $u_{I,r2}$, the surrounding rock elastic modulus *E*_3_ exhibited the highest sensitivity, followed by the cavern radius *r*_1_ and the air pressure *P*_0_. In contrast, the sensitivities of the lining elastic modulus *E*_2_, lining thickness *t*_1_, and in situ stress *P*_3_ were very low and can be considered negligible. Similarly, as shown in Figure 17b, for the radial displacement of the surrounding rock $u_{I,r3}$, except for the in situ stress *P*_3_ showing relatively high sensitivity, the sensitivity ranking of the other parameters was similar to that for the lining radial displacement. That is, *E*_3_, *r*_1_, and *P*_0_ were the three most sensitive factors, whereas the lining properties (elastic modulus and thickness) had negligible influence.

Based on the sensitivity analysis results presented in Figure 15, Figure 16 and Figure 17, it can be concluded that different factors exhibit varying degrees of influence on the mechanical responses (stress or displacement) of the structural layers (lining or surrounding rock) in a flexible membrane sealed CAES cavern, whereas certain common characteristics exist. For radial stress (Figure 15), the load conditions and cavern radius are the most critical factors, with the three most sensitive parameters being *P*_0_, *r*_1_, and *P*_3_. For the hoop stress and radial displacement (Figure 16 and Figure 17), in addition to the load conditions and cavern radius, the elastic modulus of the surrounding rock (*E*_3_) also plays a significant role. Overall, the load conditions (*P*_0_ and *P*_3_), surrounding rock elastic modulus (*E*_3_), and cavern radius (*r*_1_) were identified as the primary factors affecting the mechanical response of flexible membrane sealed CAES caverns.

To comprehensively quantify the sensitivity of each factor and establish a ranking of their relative importance, the corresponding sensitivity coefficients of each factor from Figure 15, Figure 16 and Figure 17 were summed to obtain a comprehensive sensitivity coefficient, as shown in Table 2.

As shown in Table 2, for the mechanical response of the lining (both stress and displacement), the comprehensive sensitivity of factors decreased in the order of *P*_0_, *E*_3_, *r*_1_, *P*_3_, *E*_2_, *t*_1_. For the mechanical response of the surrounding rock, the comprehensive sensitivity decreased in the order of *r*_1_, *P*_3_, *E*_3_, *P*_0_, *t*_1_, *E*_2_.

## 5. Discussion

(1) This study developed an elastic analytical model for flexible membrane sealed CAES caverns and systematically analyzed the influence of key parameters. The results indicate that internal pressure is the most critical factor controlling the hoop tensile stress of the lining and the radial deformation of the system. Appropriately increasing the lining thickness or enhancing its stiffness contributes to reducing tensile stress, while a higher elastic modulus of the surrounding rock corresponds to smaller overall deformation. Additionally, the cavern radius has a significant impact on both stress and displacement responses. These findings provide targeted engineering implications for preliminary design, including the control of operational pressure, lining selection, and reinforcement requirements under varying rock conditions.
(2) The model employs simplified assumptions, including a homogeneous and isotropic rock mass, a hydrostatic in situ stress field, and an isothermal process while also neglecting membrane stiffness. While these assumptions are applicable to shallow-buried sites with relatively low stress levels, they limit the generalizability of the model in complex geological environments. Factors such as temperature rise, interface deformation, and potential thermal fatigue behavior associated with thermal effects are excluded from the current analysis. The calculation boundary of the model was set at a sufficiently remote distance from the cavern to ensure negligible influence on the local stress field.
(3) The critical physical premise of the model—that the flexible membrane can effectively transmit internal gas pressure—has been fully validated in our previous large-scale physical model tests. This provides the physical foundation for the multi-layer structural modeling in this study. Consequently, experimental validation is not replicated herein; however, quantitative comparisons between analytical results, numerical simulations, and further experimental data will be conducted in future studies.
(4) The sensitivity analysis employs the One-At-a-Time (OAT) method, which effectively identifies dominant influencing factors but does not capture coupling effects between parameters. Future research may adopt Global Sensitivity Analysis (GSA) to obtain a more comprehensive understanding. The parameter ranges selected in this study are derived from shallow-buried cavern scenarios and existing CAES/LRC research; therefore, adjustments based on specific site conditions are necessary for practical engineering applications.

Future work will focus on incorporating thermo-mechanical coupling, in situ stress anisotropy, the Excavation Damaged Zone (EDZ), and non-linear interface interactions. Furthermore, the applicability of the model will be verified through numerical simulations and experimental tests to further refine the mechanical design method for flexible membrane sealed CAES caverns.

## 6. Conclusions

(1) Under high internal pressure, the lining and surrounding rock experience compressive radial stresses that remain below their respective strengths, so no yielding occurs. The effects of various factors can be summarized as follows: a larger cavern radius increases radial stresses in both layers; the lining’s radial stress is primarily controlled by internal pressure, whereas that of the surrounding rock depends on both internal pressure and in situ stress. Increasing the lining thickness reduces radial stress in both layers, whereas increasing the lining elastic modulus only has a minor effect. With increasing surrounding rock modulus, radial stresses in both layers rise markedly at first, then more slowly, and eventually stabilize.
(2) Increasing the internal air pressure significantly elevates the tensile hoop stresses in both the lining and surrounding rock; higher in situ stress can convert the lining’s hoop stress from tensile to compressive, thereby reducing the risk of lining cracking; increasing the cavern radius amplifies tensile hoop stress in the lining, while increasing the lining thickness can moderately reduce its peak tensile hoop stress; the increase in elastic modulus of the surrounding rock effectively reduces the lining’s tensile hoop stress. Overall, hoop stress is mainly governed by the internal pressure and surrounding rock stiffness, and the lining remains at risk of tensile cracking.
(3) Under high internal pressure, both the lining and surrounding rock undergo outward elastic radial deformation. The surrounding rock shows outward radial displacement near the lining that gradually decays with distance; under certain parameter combinations, a displacement reversal occurs, beyond which displacement shifts toward the cavern center. The effects of different factors can be summarized as follows: increasing internal pressure significantly enlarges radial deformation in both layers; higher in situ stress suppresses radial expansion and helps limit lining deformation; a larger cavern radius markedly increases the deformation magnitude, whereas greater lining thickness and stiffness substantially reduce radial displacement; increasing the surrounding rock’s elastic modulus also strongly restrains lining expansion.
(4) The loading conditions (internal air pressure *P*_0_ and in situ stress *P*_3_), the elastic modulus of the surrounding rock *E*_3_, and the cavern radius *r*_1_ were identified as the key influencing factors. A comprehensive sensitivity coefficient was proposed to quantitatively evaluate the sensitivity of each factor on the mechanical response of the CAES cavern. For the lining layer, the sensitivity of factors from high to low was ranked as *P*_0_, *E*_3_, *r*_1_, *P*_3_, *E*_2_, *t*_1_; whereas for the surrounding rock, the ranking was *r*_1_, *P*_3_, *E*_3_, *P*_0_, *t*_1_, *E*_2_.

It should be noted that in the present study, several simplifying assumptions were adopted in the theoretical analysis, such as treating the surrounding rock as a homogeneous isotropic elastic material and assuming a hydrostatic in situ stress field. Moreover, the study focused exclusively on mechanical responses, without considering associated thermodynamic processes; therefore, further research is still required to advance the engineering application of flexible membrane sealed CAES cavern technologies.

## Figures and Tables

**Table 1:** Values and variation ranges of the reference parameters.

Parameters		Units	Reference Value	Range
Geometric dimensions	Radius of the cavern, *r*_1_	m	7.5	4.0~10.0
	Thickness of the lining, *t*_1_	cm	50	30~80
Loading conditions	Internal air pressure, *p*_0_	MPa	10	6~18
	In situ stress, *p*_3_	MPa	3	1.5~12
Material properties	Elastic modulus of the lining, *E*_2_	GPa	C40	C25~C60
	Poisson’s ratio of the lining, *ν*_2_	/		
	Elastic modulus of the surrounding rock, *E*_3_	GPa	30	5~60
	Poisson’s ratio of the surrounding rock, *ν*_3_	/	0.25	Constant

Notes: This study targets shallow-buried CAES caverns (depth: 100–300 m), corresponding to an estimated vertical stress of 2.7–8.1 MPa (assuming a unit weight of 27 kN·m^−3^). Parameter selections for membrane properties and lining dimensions refer to representative studies (e.g., Ref. [13]) on abandoned mine drifts under similar conditions. Concrete elastic moduli for grades C25–C60 are adopted from GB 50010 (see Appendix A).

**Table 2:** Comprehensive sensitivity indicators of each factor of an CAES cavern.

Parameter	Comprehensive Sensitivity (Concrete Lining)	Rank	Comprehensive Sensitivity (Surrounding Rock)	Rank
*r*_1_	1.061	3	1.173	1
*t*_1_	0.069	6	0.13	5
*P*_0_	1.301	1	0.923	4
*P*_3_	0.258	4	1.096	2
*E*_2_	0.152	5	0.041	6
*E*_3_	1.128	2	1.000	3

## Data Availability

The original contributions presented in the study are included in the article. Further inquiries can be directed to the corresponding author.

## Appendix A

**Table A1:** Symbol list.

Symbol	Meaning
$p_{0}$	internal air pressure (MPa)
*t*	membrane thickness/liner thickness (cm)
$r_{1}$	tunnel cavity radius/inner radius (m)
$\sigma_{r1}$	radial stress in membrane (MPa)
$\sigma_{\theta 1}$	hoop stress/circumferential stress (MPa)
$\tau_{r\theta 1}$	shear stress (MPa)
*E*_1_	Young’s modulus of membrane (GPa)
*ν*_1_	Poisson’s ratio of membrane
${\left(u_{r1}\right)}_{\max}$	maximum radial displacement (cm)
Δ	gap/clearance between membrane and lining
*P*_1_	inner pressure on membrane (MPa)
*P*_2_	outer pressure on membrane (MPa)
*P*_3_	far-field stress/in situ stress (MPa)
*r*	radial coordinate (m)
*i*	layer index
*r*_i−1_, *r*_i_	inner/outer radius of layer *i* (m)
*r*_3_	truncation boundary radius (far radius) (m)
*A*ᵢ, *C*ᵢ	integration constants
*A*_2_, *C*_2_	constants for concrete lining
*A*_3_, *C*_3_	constants for rock mass
*c*	coefficient vector
*B*	coefficient matrix/relationship matrix
y	load vector
*t*_1_	concrete lining thickness(cm)
*E*_2_	Young’s modulus of concrete lining (GPa)
*ν*_2_	Poisson’s ratio of lining concrete
*E*_3_	Young’s modulus of surrounding rock (GPa)
*ν*_3_	Poisson’s ratio of surrounding rock
$\sigma_{r2}$	radial stress in concrete lining (MPa)
$\sigma_{r3}$	radial stress in surrounding rock (MPa)
$\sigma_{I,\theta 2}$	hoop stress in lining (MPa)
$\sigma_{I,\theta 3}$	hoop stress in rock (MPa)
$u_{I,r2}$	radial displacement of lining (mm)
$u_{I,r3}$	radial displacement of rock (mm)
$\beta_{i}$	load-sharing ratio of layer *i*
$\sigma_{i}$	radial stress difference of layer *i* (MPa)
p	pressure difference (p_0_–p_3_) (MPa)
X	parameter vector
Xmin	lower bound of parameter
Xmax	upper bound of parameter
ξ	uniform random number (0–1)
*f*	response function

**Table A2:** Elastic modulus of different grades of concrete.

Grade	C25	C30	C35	C40	C45	C50	C55	C60
Ec (GPa)	28.0	30	31.5	32.5	33.5	34.5	35.5	36.0

Notes: GB 50010 recommends that the Poisson’s ratio of concrete can be taken as v ≈ 0.20 (commonly used value).
